# Supplementary material for: Development of Cell Permeable NanoBRET Probes for the Measurement of PLK1 Target Engagement in Live Cells
Source: bioRxiv. 2023 Mar 7:2023.02.25.529946. Preprint. [Version 3] doi: 10.1101/2023.02.25.529946 (PMC9980182; doi:10.1101/2023.02.25.529946)

Supplementary Material

Development of Cell Permeable NanoBRET Probes for the Measurement of PLK1 Target Engagement in Live Cells

Xuan Yang[1], Jeffery L. Smith[1], Michael T. Beck[2], Jennifer M. Wilkinson[2], Ani Michaud[2], James D. Vasta[2], Matthew B. Robers[2], and Timothy M. Willson[1]*

[1] Structural Genomics Consortium, UNC Eshelman School of Pharmacy, University of North Carolina at Chapel Hill, Chapel Hill, NC 27599, USA

[2] Promega Corporation, 2800 Woods Hollow Road, Fitchburg, WI 53711, USA

* Correspondence: tim.willson@unc.edu

Figure S1: Tracer titration of probe **11** on NLuc-PLK2 and NLuc-PLK3

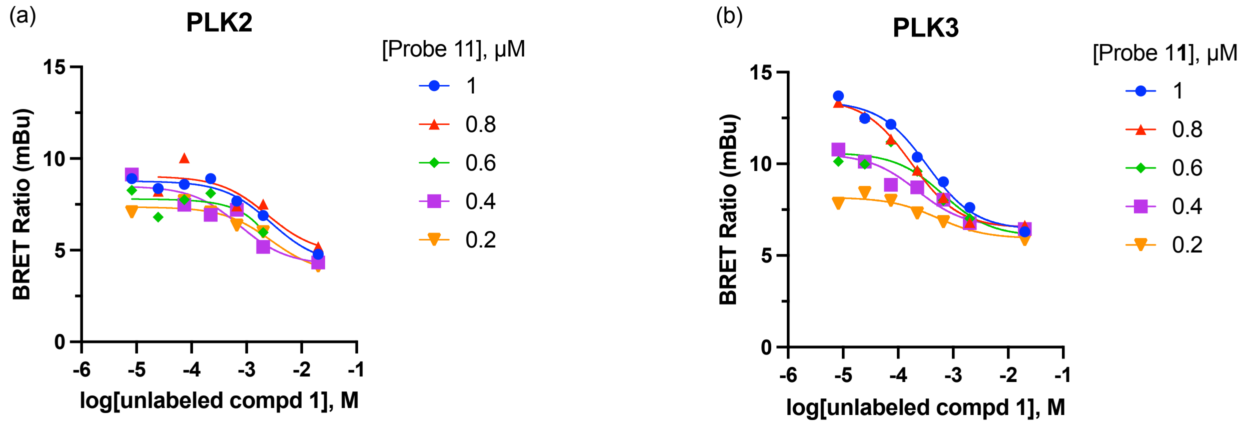

BRET ratios of (a) NLuc-PLK2 and (b) NLuc-PLK3 using multiple concentration of probe **11** with increasing doses of intermediate **1** as a competitor. The 1 μM concentration of probe **11** was selected for use in the PLK2 and PLK3 NanoBRET assays.

^{1}H and ^{13}C NMR Spectra of intermediate **1**, probe **10**, and probe **11**:

^{1}H NMR spectrum of intermediate **1** in MeOD

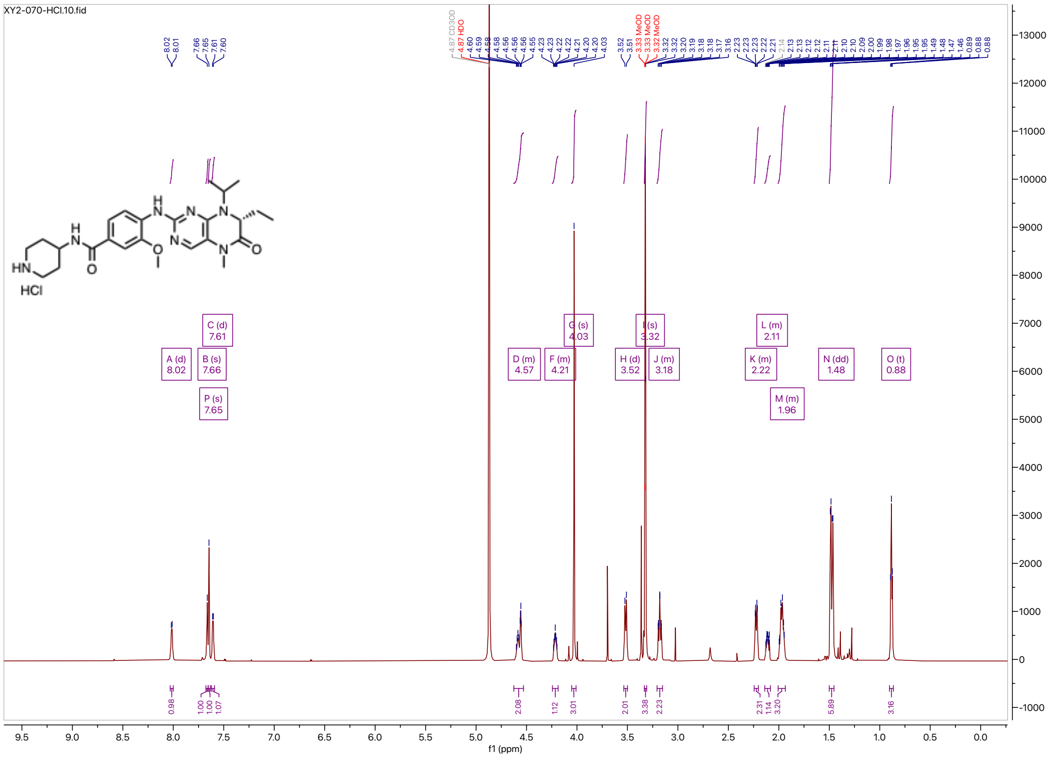

^{13}C NMR spectrum of intermediate **1** in MeOD

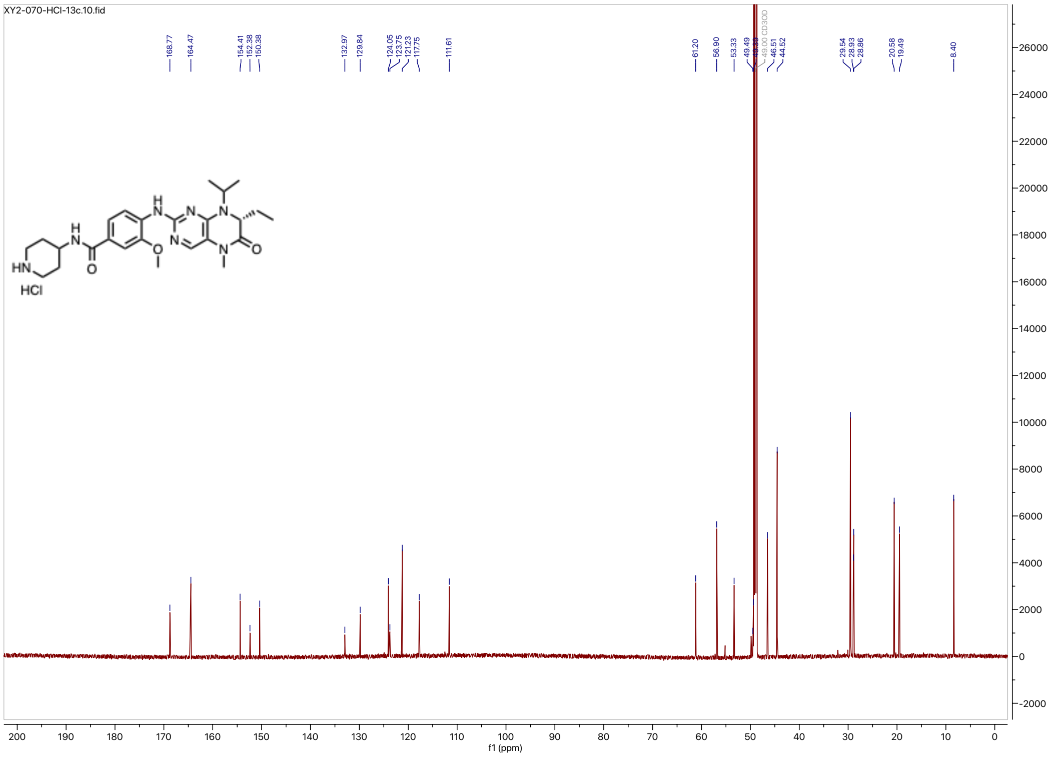

^{1}H NMR spectrum of probe **10** in MeOD

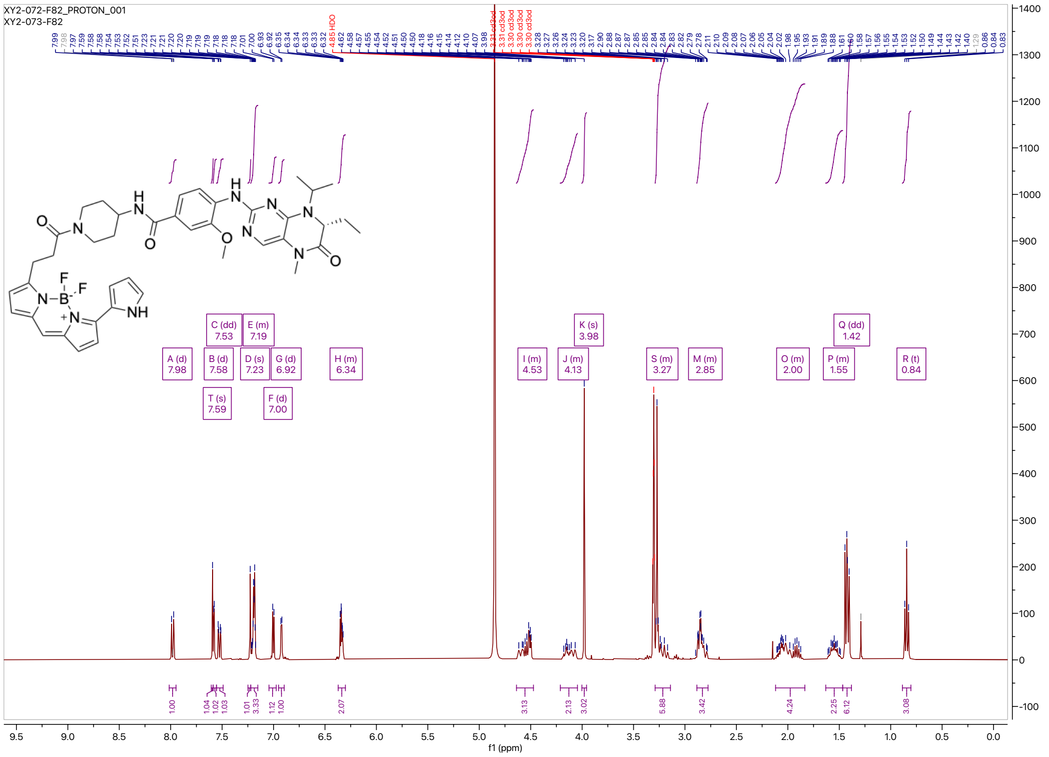

^{13}C NMR spectrum of probe **10** in MeOD

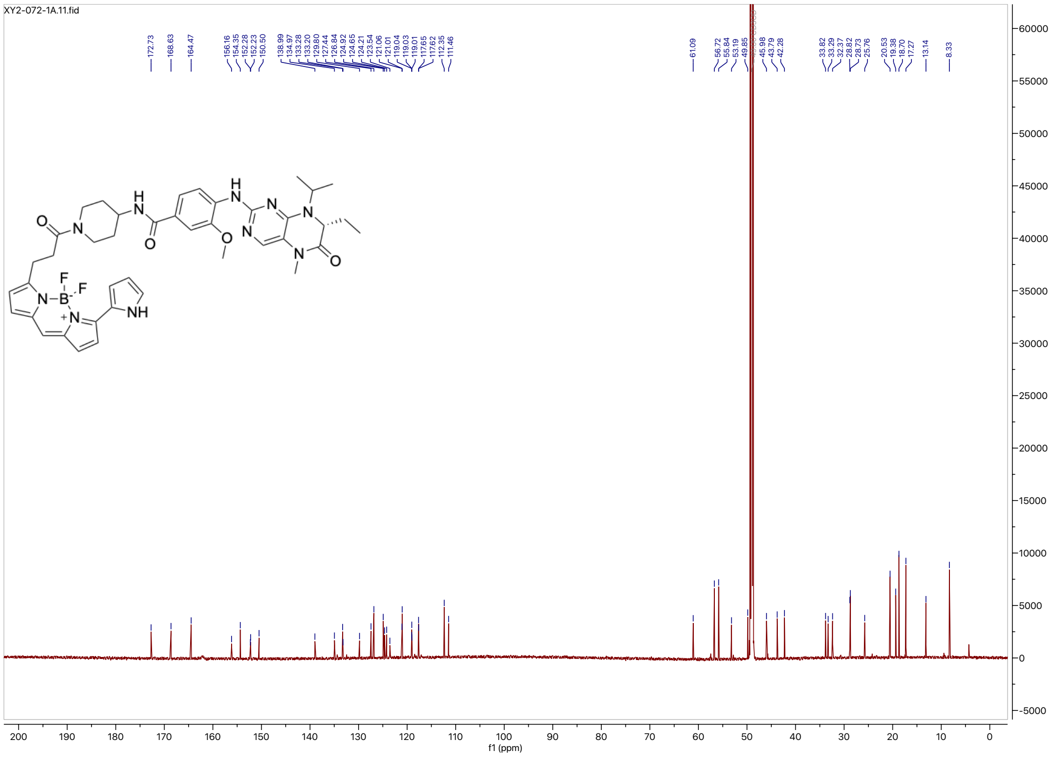

^{1}H NMR spectrum of probe **11** in MeOD

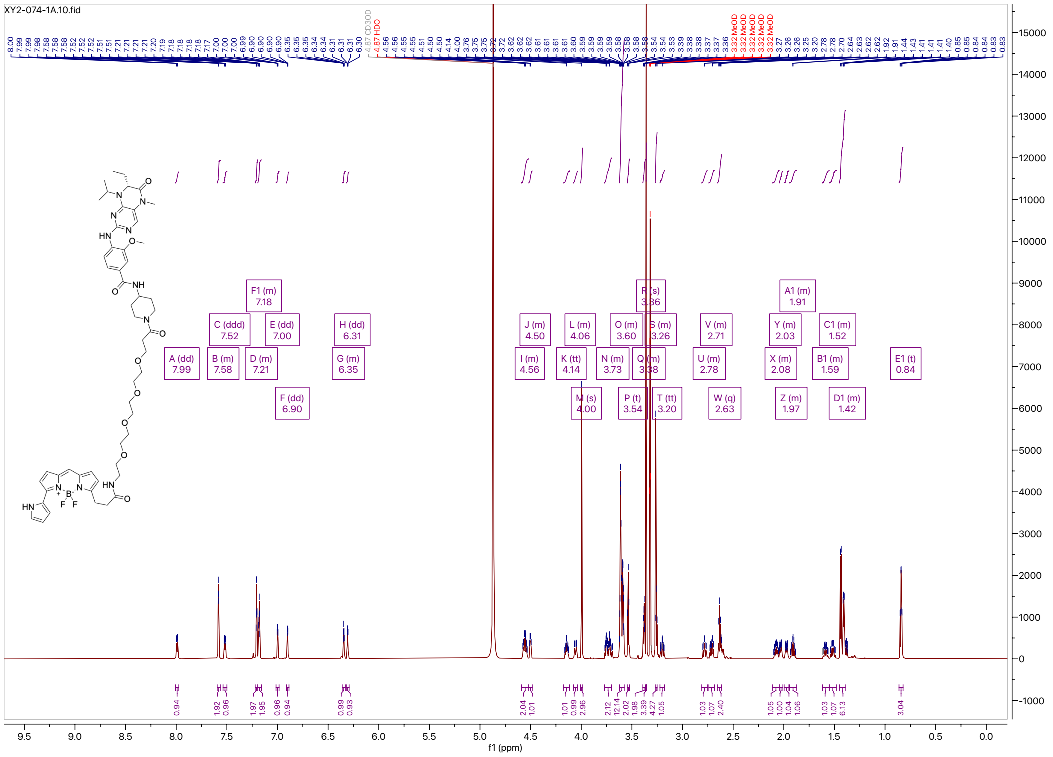

^{13}C NMR spectrum of probe **11** in MeOD

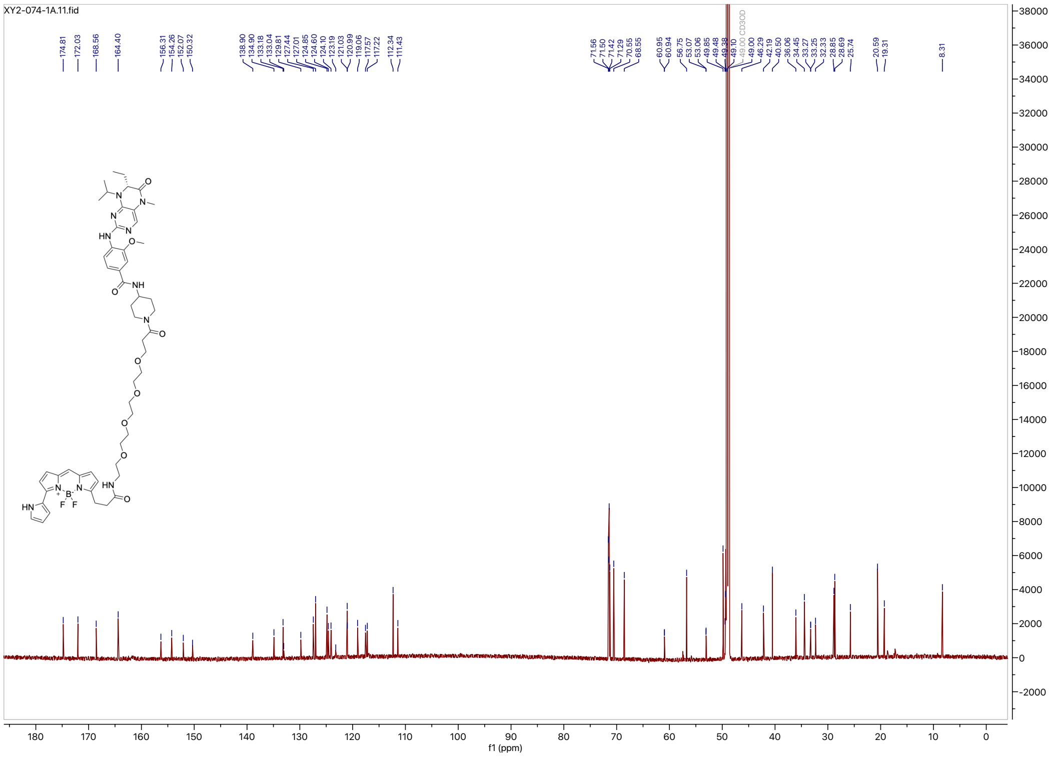

HSQC spectrum of probe **11** in MeOD

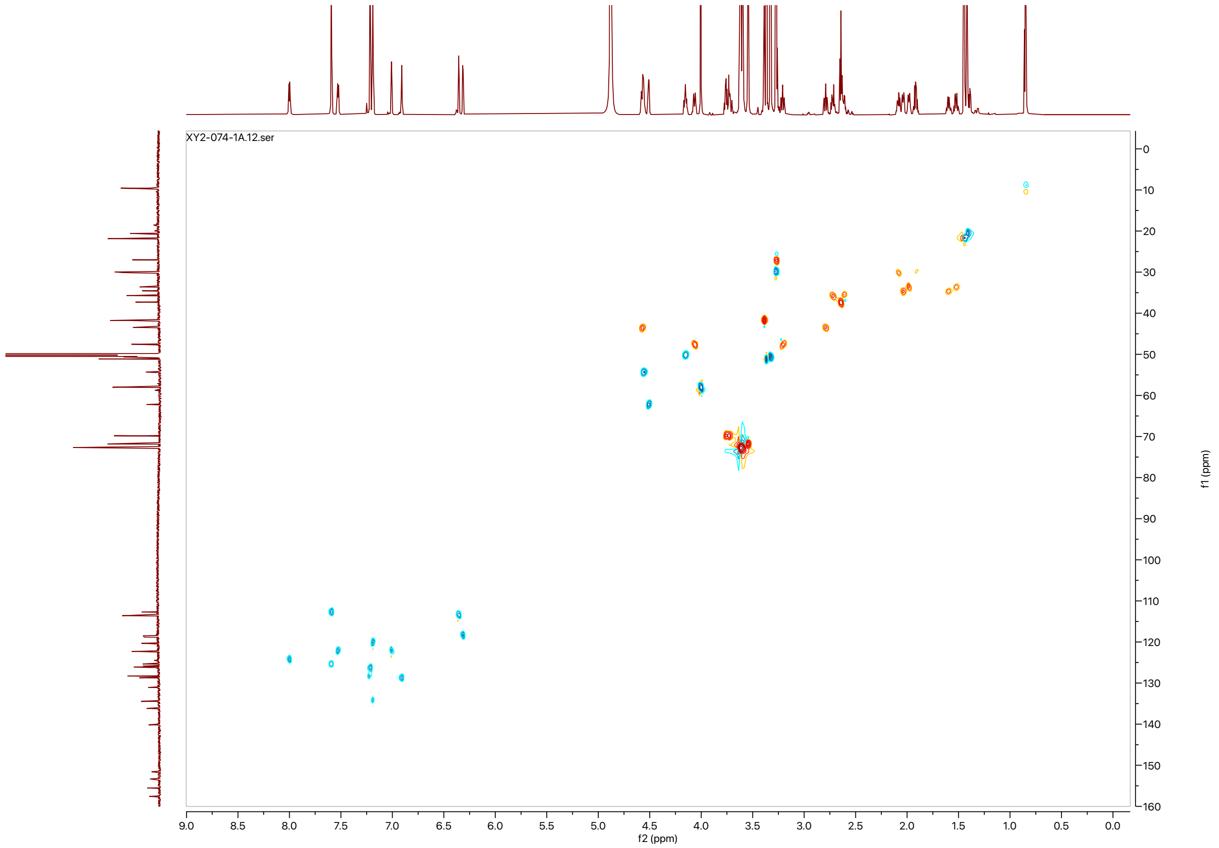

Supplement: Supplement 1 — Figure S1: Tracer titration of probe 11 on NLuc-PLK2 and NLuc-PLK3. 1H and 13C NMR Spectra of intermediate 1, probe 10, and probe 11. [file media-1.pdf]
